# Ethanologenesis and respiration in a pyruvate decarboxylase-deficient *Zymomonas mobilis*

**DOI:** 10.1186/s13104-021-05625-5

**Published:** 2021-05-28

**Authors:** Reinis Rutkis, Inese Strazdina, Zane Lasa, Per Bruheim, Uldis Kalnenieks

**Affiliations:** 1 Institute of Microbiology and Biotechnology, University of Latvia, Riga, Latvia; 2 Department of Biotechnology and Food Science, NTNU Norwegian University of Science and Technology, Trondheim, Norway

**Keywords:** *Zymomonas mobilis*, Pyruvate decarboxylase, Respiratory chain, Alcohol dehydrogenase, Acetaldehyde, Pyruvate

## Abstract

**Objective:**

*Zymomonas mobilis* is an alpha-proteobacterium with a rapid ethanologenic pathway, involving Entner–Doudoroff (E–D) glycolysis, pyruvate decarboxylase (Pdc) and two alcohol dehydrogenase (ADH) isoenzymes. Pyruvate is the end-product of the E–D pathway and the substrate for Pdc. Construction and study of Pdc-deficient strains is of key importance for *Z*.* mobilis* metabolic engineering, because the pyruvate node represents the central branching point, most novel pathways divert from ethanol synthesis. In the present work, we examined the aerobic metabolism of a strain with partly inactivated Pdc.

**Results:**

Relative to its parent strain the mutant produced more pyruvate. Yet, it also yielded more acetaldehyde, the product of the Pdc reaction and the substrate for ADH, although the bulk ADH activity was similar in both strains, while the Pdc activity in the mutant was reduced by half. Simulations with the kinetic model of *Z*.* mobilis* E-D pathway indicated that, for the observed acetaldehyde to ethanol production ratio in the mutant, the ratio between its respiratory NADH oxidase and ADH activities should be significantly higher, than the measured values. Implications of this finding for the directionality of the ADH isoenzyme operation in vivo and interactions between ADH and Pdc are discussed.

**Supplementary Information:**

The online version contains supplementary material available at 10.1186/s13104-021-05625-5.

## Introduction

*Zymomonas mobilis* is a Gram-negative, facultatively anaerobic alpha-proteobacterium, known for its highly efficient ethanol pathway, which is composed of E–D glycolysis in combination with pyruvate decarboxylase (Pdc) and two alcohol dehydrogenase isoenzymes—ADH I and ADH II [1, 2]. This bacterium also possesses an active respiratory chain with low energy-coupling efficiency [3–6]. Apart from bioethanol, the rapid catabolism of *Z*.* mobilis* offers several capacities to engineer this bacterium for alternative products [7]. The pyruvate node of the E–D pathway represents the branching point, at which engineered pathways divert from the ethanol synthesis. Pyruvate is the end-product of the E–D pathway and the substrate for pyruvate decarboxylase. Hence, in order to turn carbon flux from ethanol synthesis to alternative products, it is essential to inactivate Pdc.

Inactivation of Pdc is anticipated to cause accumulation of pyruvate and a redox disbalance, due to a shortage of acetaldehyde for the ADH reaction. A seemingly straightforward way to resolve the redox disbalance would be to regenerate NAD by respiratory chain with simultaneous production and export of pyruvate instead of ethanol. However, current evidence shows that *pdc* is essential for *Z*.* mobilis* growth both under anaerobic and aerobic conditions [8]. Insertional inactivation of *pdc* by homologous recombination has resulted in ‘leaky’ knock-out mutants [7, 9–12], which retain also the wild-type gene copy. Liu et al. [8] succeeded to completely delete the chromosomal copy of *pdc*, while simultaneously ensuring regulated expression of *pdc* from a plasmid vector. Growth and product synthesis of these partial *pdc* knock-out strains were studied under anaerobic or microaerobic conditions. Lower specific rates of growth, decreased glucose consumption and ethanol production, elevated accumulation of pyruvate and an increase of lactate and glycerol production were reported [8, 9]. Here we focussed on aerobic growth of the partial knock-out strain. Somewhat unexpectedly, under oxic conditions an increase of acetaldehyde occurred instead of pyruvate becoming the major catabolic product. We provide a model simulation and analysis of these aerobic effects.

## Main text

### Methods

#### *Zymomonas mobilis* strains and cultivation

Strain Zm6 (ATCC 29191) and its mutant derivative *pdc*, carrying a tetracycline resistance marker inserted in the chromosomal copy of its *pdc* gene [12], were studied. The mutant strain was only partially Pdc-deficient. PCR reaction on its chromosomal DNA template amplified two products, one of which was identified by sequencing as the disrupted gene copy carrying the antibiotic resistance insert, but the other one was the intact gene copy (see Additional file 1: Figures S1, S2). The *pdc* strain retained 50–60% of the wild-type Pdc activity [12].

Three aerobic batch cultivations for each *Z*.* mobilis* strain were carried out in multi parallel Applikon my-Control bioreactors (500 mL working volume) at 30 °C operated with BioExpert V2 software. The gas flow was kept at 0.5 L min^−1^ and agitation at 200 rpm. Growth medium contained 50 g L^−1^ glucose, 5 g L^−1^ yeast extract and mineral salts, as described earlier [13]. Samples were taken every 2 h for measurement of substrate and product concentrations. MS analysis of the fermenter outlet gas was carried out by Thermoscientific PrimaBT Benchtop MS mass spectrometer, continuously monitoring the concentration of O_2_, CO_2_, acetaldehyde and ethanol in the gas phase.

#### Analytical assays

Ethanol and glucose concentrations were measured by HPLC [14]. Acetaldehyde concentration in the liquid phase was measured enzymatically with Megazyme acetaldehyde kit K-ACHYD (based on NADH generation during acetaldehyde oxidation to acetate by aldehyde dehydrogenase), following manufacturer’s instructions. Pyruvate was monitored with lactate dehydrogenase assay [12]. Samples for NAD(P)/NAD(P)H measurements were taken at late exponential growth phase, and analysed by LC/tandem MS [15]. The total ADH activity in ultrasonic cell-free extracts was assayed spectrophotometrically, by monitoring NADH absorbance increase at 340 nm after ethanol addition [16]. ADH I contribution to the total ADH activity was calculated from assays with butanol as the substrate [17].

#### Metabolic modeling

The backbone of kinetic model of the *Z*.* mobilis* E–D pathway that was used in this study was described and validated previously [18] (see BioModels database ID: MODEL 1409050000). Later, for modeling the interplay between the E–D pathway and respiration, acetaldehyde export reaction was introduced, and NADH withdrawal by respiratory chain was added as a simple sink reaction [6]. The model used by Rutkis et al. [6] served as the basis for simulations in the present work (see BioModels database ID: MODEL 2008060001). As previously, kinetic modeling and simulations were carried out using COPASI software.

### Results and discussion

#### Partial inactivation of Pdc results in a rise of acetaldehyde yield

**Fig. 1:**
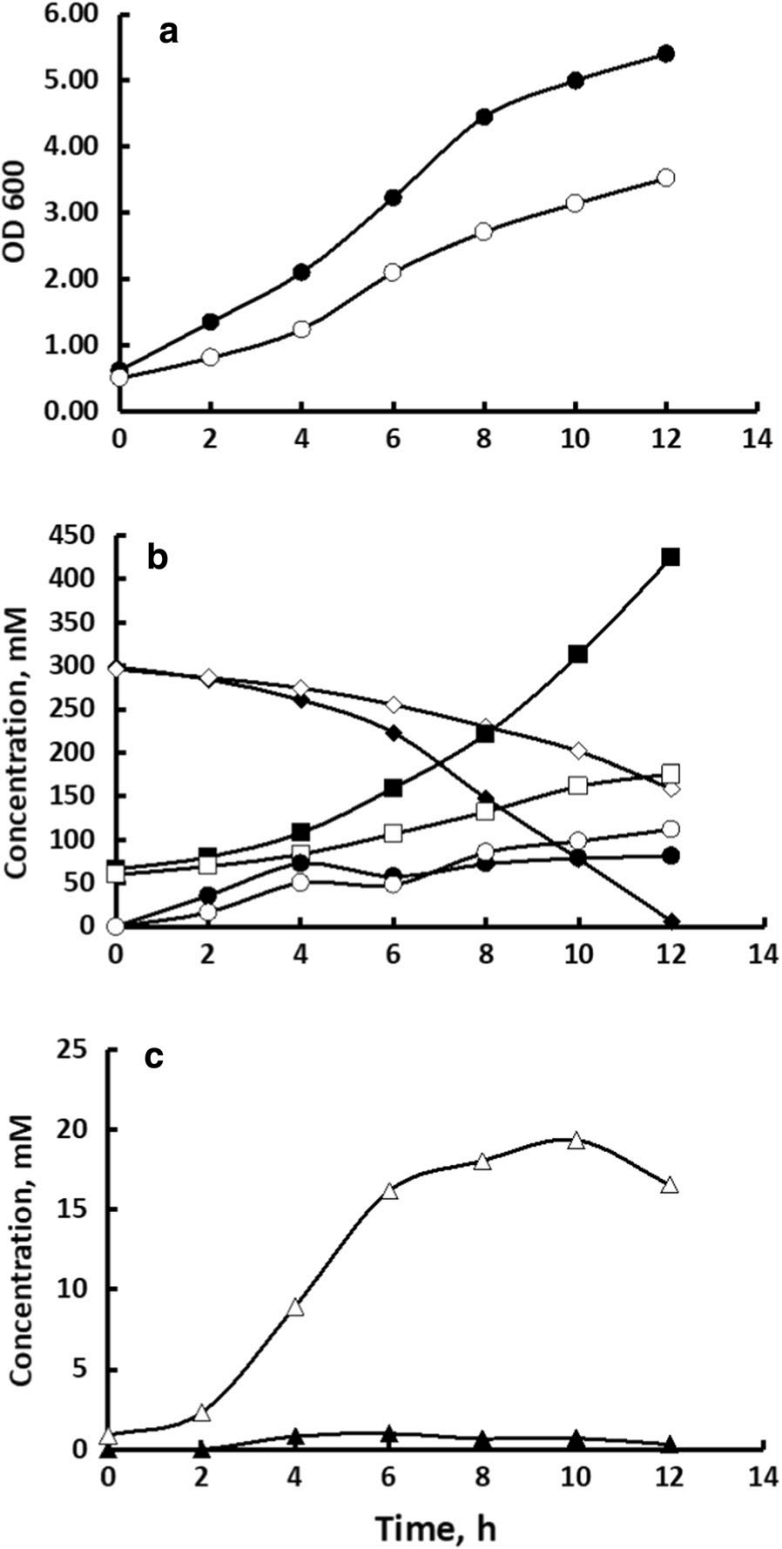
Aerobic batch cultivation of Zm6 (closed symbols) and *pdc* (open symbols). **A** growth curves; **B** the time course of glucose consumption (diamonds), ethanol production (squares), and acetaldehyde production (circles); **C** pyruvate production. Data on acetaldehyde and ethanol are corrected for evaporation, based on MS-monitoring of the fermenter outlet gas

Both strains were cultivated in aerobic batch mode. The Pdc-deficient strain showed slower specific growth rate and slower glucose consumption (Table 1, Fig. 1). Both the specific growth and CO_2_ production rates (µ and Q_CO2_, respectively) of the *pdc* strain were approximately 80% of the wild type levels. As expected, the *pdc* strain produced more pyruvate than Zm6 (Fig. 1C). During 12 h of cultivation, pyruvate accumulated up to 16 mM concentration in the mutant culture, while barely 1 mM concentration was reached in Zm6. This falls within the concentration range recently reported for the anaerobic (microaerobic) Pdc-deficient ZM4-derived mutant cultures [8, 9]. Nevertheless, pyruvate aerobically still represented a minor byproduct of *pdc*; its yield was just 0.15 mol per mole of glucose (Table 1). Instead of turning pyruvate into the dominant product, under aerobic culture conditions partial inactivation of Pdc resulted in a shift of the major catabolic product yields downstream the Pdc reaction. As shown in Fig. 1B, *pdc* strain consumed glucose slower, and accordingly, produced much less ethanol, yet the amount of acetaldehyde that it generated, was comparable or even slightly higher than in Zm6. Intuitively one would expect the opposite—that a decrease of the pyruvate decarboxylase activity would reduce the acetaldehyde yield, since acetaldehyde is the product of the Pdc reaction, while at the same time, ADH in the *pdc* strain operates in the direction of net ethanol synthesis. Yet, despite of having only half of the wild type Pdc activity, the *pdc* strain produced acetaldehyde with a yield nearly twice of Zm6 (Table 1). Importantly, the sum of the measured molar ratios [ethanol/CO_2_] and [acetaldehyde/CO_2_] was very close to unity in both strains, indicating that carbon recovery of the products of pyruvate degradation in our cultivations was near to 100%. The molar ratio of acetaldehyde to ethanol, as estimated from the Table 1 data, differed considerably: it was 0.38 for the wild type and 0.91 for *pdc*.

**Table 1:** Kinetic and stoichiometric parameters of aerobic batch cultivations. Specific rates (Q) are presented as [mmoles (g h)^−1^], ADH activities as [U mg prot^−1^], and yield values (Y) and NAD(P)(H) ratios are given as molar ratios. Means of three cultivation experiments are presented, with standard deviation values in brackets. Statistical significance of differences between the strains: *P < 0.05; **P < 0.01

	Zm6	*pdc*
µ [h^–1^]	0.27 (± 0.03)	0.23 (± 0.01)
Q_gluc_	33.1 (± 5.3)	28.4 (± 1.7)
Q_CO2_*	57.5 (± 6.0)	45.1 (± 3.5)
Q_O2_	24.2 (± 1.8)	20.3 (± 1.6)
Y_pyr/gluc_*	0.03 (± 0.02)	0.15 (± 0.06)
Y_EtOH/gluc_	1.29 (± 0.11)	1.01 (± 0.24)
Y _EtOH/CO2_**	0.72 (± 0.05)	0.52 (± 0.03)
Y_acetald/gluc_*	0.49 (± 0.21)	0.92 (± 0.12)
Y_acetald/CO2_*	0.27 (± 0.12)	0.49 (± 0.08)
ADH I	3.17 (± 0.13)	3.41 (± 0.22)
ADH II	1.25 (± 0.23)	0.82 (± 0.19)
[NADH/NAD]	0.168 (± 0.011)	0.184 (± 0.004)
[NADH/NADP]	0.047 (± 0.008)	0.043 (± 0.009)

Previous stoichiometric analysis of *Z*.* mobilis* catabolism in combination with screening of *Z*.* mobilis* mutant phenotypes [19] has shown that an increase in acetaldehyde yield could be attained by redistribution of the reducing equivalents flux, directing more NADH to the respiratory chain and concomitantly, diminishing NADH supply to the ADH reaction. Such a redistribution could happen, if the ADH activity was decreased, and/or the activity of the electron transport chain was elevated.

#### Model simulation of aerobic product yields does not conform to the experimentally measured ratio between respiratory oxidase and alcohol dehydrogenase activities

We applied the E–D kinetic model for simulation of the relationship between the ratio of produced acetaldehyde to produced ethanol and the ratio between the Vmax of NADH oxidase (Vmax_Ndh_) and that of ADH (Vmax_ADH_, in the direction of acetaldehyde reduction). For Zm6 the model parameters were taken as previously [6, 18]. In order to adjust the model to the *pdc* strain: (i) Vmax value of pyruvate decarboxylase in the original model was set at 60% of that of the Zm6, and (ii) the rate of the lumped reaction describing the metabolic ATP consumption/dissipation was set at 80% of the respective Zm6 value. As result, simulation produced around 80% of the glycolytic rate of wild type, in a good agreement with our experimental data for the *pdc* strain (Table 1).

The in vivo Vma_Ndh_ was deduced from the culture oxygen consumption rates (Table 1). From the oxygen consumption data, NADH oxidation rate for both strains was calculated to be around 1.2–1.3 U mg prot^−1^. For calculations we assumed that: (i) NADH oxidation by Ndh represented the major fraction of *Z*.* mobilis* oxygen consumption [13], and (ii) two moles of NADH were oxidised per mole of O_2_ [20]. In both strains, the NADH/NAD ratio was in the range of 0.16–0.18 (Table 1). Given the *Z*.* mobilis* intracellular NAD(H) pool about 5 mM [21], that implied the intracellular NADH levels close to 1 mM. Since the Km of *Z*.* mobilis* Ndh is 60 µM [22], Ndh in both strains under these conditions should be operating at its near-maximum rate. Hence, the Vmax_Ndh_ was approximated as 1.2–1.3 U mg prot^−1^.

**Fig. 2:**
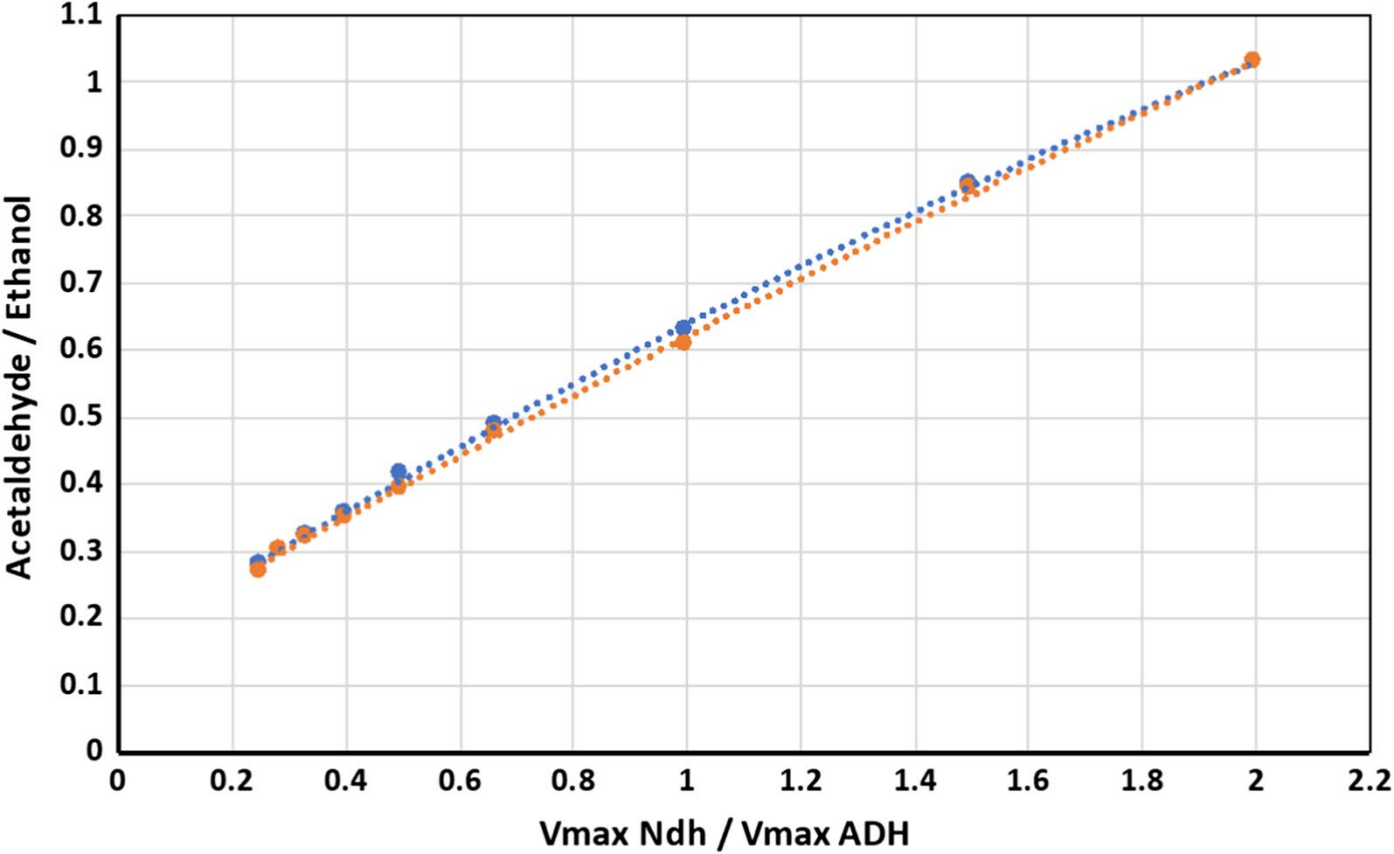
Model simulation data on the relation between the ratio of the Vmax values of NADH oxidase (Ndh) and ADH and the acetaldehyde to ethanol molar ratio. Orange symbols, strain Zm6; blue symbols, strain *pdc*

Simulation experiments were performed by stepwise variation of the sum of Vmax values of both ADH isoenzymes in the range between 0 and 6 U mg prot^−1^. The ratio between the Vmax_Ndh_ and Vmax_ADH_, and the corresponding ratio between the steady-state rates of acetaldehyde and ethanol production were calculated for each simulation run. Results of simulation experiments with Vmax ratios between 0.25 and 2.0 are depicted in the Fig. 2. According to Fig. 2, the shift of product ratio from 0.38 to 0.91 would correspond to an increase of the [Vmax_Ndh_/Vmax_ADH_] almost by a factor of 4; namely, from around 0.45 to 1.65–1.70.

However, no significant differences were found between both strains with respect to the ADH isoenzyme activities in cell-free extracts. Both ADH activities presented in Table 1 were measured in the direction of ethanol oxidation at pH 8.2. For estimation of the intracellular Vmax_ADH_ in the direction of acetaldehyde reduction at physiological pH values, we used kinetic data from the study of Neale et al. [23], assuming the intracellular pH in *Z*.* mobilis* close to 6.5 [24]. The Vmax values for acetaldehyde reduction thus estimated, were about 7 U mg prot^−1^ for ADH I and 0.2 U mg prot^−1^ for ADH II. As result, we arrived at a Vmax_Ndh_/Vmax_ADH_ ratio in the range of 0.17–0.18 for both strains. This was somewhat less than our model predicted for Zm6 (for acetaldehyde to ethanol ratio 0.38 the Vmax ratio should be around 0.45; see Fig. 2). Yet, for the *pdc* strain there was a huge discrepancy, since for the acetaldehyde to ethanol ratio 0.91 the model-predicted Vmax ratio should reach 1.65–1.70.

#### Is the partial disruption of Pdc affecting the intracellular function of alcohol dehydrogenases?

The estimated Vmax_Ndh_/Vmax_ADH_ ratio of 0.17–0.18 is based on an implicit assumption that in respiring cells both ADH isoenzymes function exclusively in the direction of ethanol synthesis. However, our previous study of aerobic continuous cultures of the wild type (Zm6) and an ADH II-deficient mutant has indicated that the ADH isoenzymes might operate in opposite directions, forming an ‘ethanol cycle’ [16, 25]. Some part of ADH in the cytosol operates in oxidative direction, which implies the intracellular net ethanol-synthesizing activity to be less than the sum of activities estimated from the measurements in cell-free extract. Taking that into account brings the actual Vmax_Ndh_/Vmax_ADH_ ratio estimate closer to the model prediction. Accordingly, the large discrepancy between experiment and simulation for the *pdc* strain would mean that in the mutant more of ADH is catalyzing in vivo oxidation of ethanol, than in the parent strain. We hypothesize that in *Z*.* mobilis* cytosol Pdc and ADH physically interact, and acetaldehyde gets channelled between their active sites, like recently demonstrated for the AdhE of *E. coli* [26]. Acetaldehyde is a toxic compound, and its channelling would help to keep its intracellular pool low. In the *pdc* strain this channelling might be disturbed, because some part of ADH apparently is left without its functional Pdc counterpart, and might be contributing mostly to ethanol oxidation.

#### Limitation

Although the metabolic channelling hypothesis provides a plausible explanation of the observed effect of the Pdc deficiency on acetaldehyde production, a direct experimental evidence is required to support the proposed mechanism.

## Supplementary Information

**Additional file 1:** The sequence, primers and PCR products of *pdc* gene in the parent and mutant strain.

## Data Availability

The kinetic model used for simulations is deposited un the BioModels database; ID: MODEL 2008060001. The datasets and strains used in the present study are available from the corresponding author upon request.

## Abbreviations

E–D: Entner Doudoroff pathway
ADH: Alcohol dehydrogenase
Pdc: Pyruvate decarboxylase
Ndh: Type II respiratory NADH dehydrogenase

## References

[1] Rogers PL, Lee KJ, Skotnicki ML, Tribe DE (1982). Ethanol production by *Zymomonas mobilis*. Adv Biochem Eng.

[2] Sprenger GA (1996). Carbohydrate metabolism in *Zymomonas mobilis*: a catabolic highway with some scenic routes. FEMS Microbiol Lett.

[3] Strohdeicher M, Neuß B, Bringer-Meyer S, Sahm H (1990). Electron transport chain of *Zymomonas mobilis*. Interaction with the membrane-bound glucose dehydrogenase and identification of ubiquinone 10. Arch Microbiol.

[4] Kalnenieks U, Galinina N, Bringer-Meyer S, Poole RK (1998). Membrane d-lactate oxidase in *Zymomonas mobilis*: evidence for a branched respiratory chain. FEMS Microbiol Lett.

[5] Rutkis R, Galinina N, Strazdina I, Kalnenieks U (2014). The inefficient aerobic energetics of *Zymomonas mobilis*: identifying the bottleneck. J Basic Microbiol.

[6] Rutkis R, Strazdina I, Balodite E, Lasa Z, Galinina N, Kalnenieks U (2016). The low energy-coupling respiration in *Zymomonas mobilis* accelerates flux in the Entner–Doudoroff pathway. PLoS ONE.

[7] Wang X, He Q, Yang Y, Wang J, Haning K, Hu Y, Wu B, He M, Zhang Y, Bao J (2018). Advances and prospects in metabolic engineering of *Zymomonas mobilis*. Metab Eng.

[8] Liu Y, Ghosh IN, Martien J, Zhang Y, Amador-Noguez D, Landick R (2020). Regulated redirection of central carbon flux enhances anaerobic production of bioproducts in *Zymomonas mobilis*. Metabol Eng.

[9] Zhao X, Rogers PL, Kwon EE, Jeong SC, Jeon YJ (2015). Growth characteristics of a pyruvate decarboxylase mutant strain of *Zymomonas mobilis*. J Life Sci.

[10] Shui Z, Wang J, Qin H, Wu B, Tan F, He M (2015). Construction and preliminary fermentation of succinate-producing recombinant ethanologenic *Zymomonas mobilis*. Chinese J Appl Environ Biol.

[11] Yang S, Mohagheghi A, Franden MA, Chou YC, Chen X, Dowe N, Himmel ME, Zhang M (2016). Metabolic engineering of *Zymomonas mobilis* for 2,3-butanediol production from lignocellulosic biomass sugars. Biotechnol Biofuels.

[12] Balodite E, Strazdina I, Martynova J, Galinina N, Rutkis R, Lasa Z, Kalnenieks U (2019). Translocation of *Zymomonas mobilis* pyruvate decarboxylase to periplasmic compartment for production of acetaldehyde outside the cytosol. MicrobiologyOpen.

[13] Kalnenieks U, Galinina N, Strazdina I, Kravale Z, Pickford JL, Rutkis R, Poole RK (2008). NADH dehydrogenase deficiency results in low respiration rate and improved aerobic growth of *Zymomonas mobilis*. Microbiology.

[14] Strazdina I, Balodite E, Lasa Z, Rutkis R, Galinina N, Kalnenieks U (2018). Aerobic catabolism and respiratory lactate bypass in Ndh-negative *Zymomonas mobilis*. Metabol Eng Commun.

[15] Røst L, Shafaei A, Fuchino K, Bruheim P (2020). Zwitterionic HILIC tandem mass spectrometry with isotope dilution for rapid, sensitive and robust quantification of pyridine nucleotides in biological extracts. J Chromatography B.

[16] Kalnenieks U, Galinina N, Toma MM, Pickford JL, Rutkis R, Poole RK (2006). Respiratory behaviour of a *Zymomonas mobilis adhB*: *kan*^*r*^ mutant supports the hypothesis of two alcohol dehydrogenase isoenzymes catalysing opposite reactions. FEBS Lett.

[17] Kinoshita S, Kakizono T, Kadota K, Das K, Taguchi H (1985). Purification of two alcohol dehydrogenases from *Zymomonas mobilis* and their properties. Appl Microbiol Biotechnol.

[18] Rutkis R, Kalnenieks U, Stalidzans E, Fell DA (2013). Kinetic modelling of the *Zymomonas mobilis* Entner–Doudoroff pathway: insights into control and functionality. Microbiology.

[19] Kalnenieks U, Balodite E, Strähler S, Strazdina I, Rex J, Pentjuss A, Fuchino K, Bruheim P, Rutkis R, Pappas KM (2019). Improvement of acetaldehyde production in *Zymomonas mobilis* by engineering of its aerobic metabolism. Front Microbiol.

[20] Bringer S, Finn RK, Sahm H (1984). Effect of oxygen on the metabolism of *Zymomonas mobilis*. Arch Microbiol.

[21] de Graaf AA, Striegel K, Wittig RM, Laufer B, Schmitz G, Wiechert W, Sprenger GA, Sahm H (1999). Metabolic state of *Zymomonas mobilis* in glucose-, fructose-, and xylose-fed continuous cultures as analysed by ^13^C- and ^31^P-NMR spectroscopy. Arch Microbiol.

[22] Kim YJ, Song KB, Rhee SK (1995). A novel aerobic respiratory chain-linked NADH oxidase system in *Zymomonas mobilis*. J Bacteriol.

[23] Neale AD, Scopes RK, Kelly JM, Wettenhall RE (1986). The two alcohol dehydrogenases of *Zymomonas mobilis*. Purification by differential dye ligand chromatography, molecular characterisation and physiological roles. Eur J Biochem.

[24] Barrow KD, Collins JG, Norton RS, Rogers PL, Smith GM (1984). ^31^P nuclear magnetic resonance studies of the fermentation of glucose to ethanol by *Zymomonas mobilis*. J Biol Chem.

[25] Kalnenieks U, Galinina N, Toma MM, Marjutina U (2002). Ethanol cycle in an ethanologenic bacterium. FEBS Lett.

[26] Pony P, Rapisarda C, Terradot L, Marza E, Fronzes R (2020). Filamentation of the bacterial bifunctional alcohol/aldehyde dehydrogenase AdhE is essential for substrate channeling and enzymatic regulation. Nat Commun.

