# Supplementary material for: Ethanologenesis and respiration in a pyruvate decarboxylase-deficient Zymomonas mobilis
Source: BMC Res Notes. 2021 May 28;14:208. doi: 10.1186/s13104-021-05625-5 (PMC8161578; doi:10.1186/s13104-021-05625-5)
Supplement: Supplementary file 1 — Additional file 1: The sequence, primers and PCR products of pdc gene in the parent and mutant strain. [file 13104_2021_5625_MOESM1_ESM.docx]

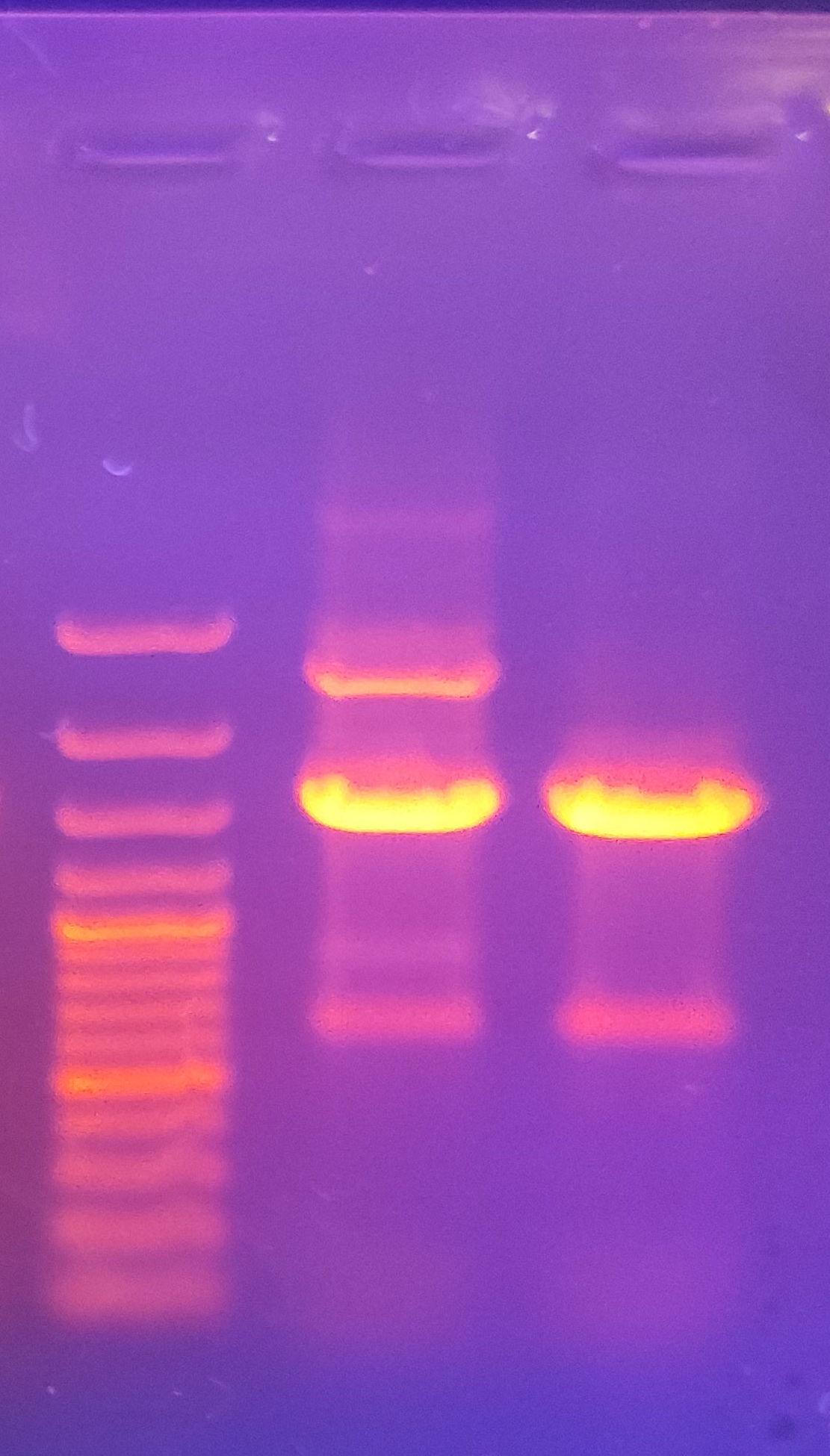


*pdc* Zm6

bp:

3000

2000

1500

**Figure S1. PCR products with primers pdc1 and pdc2 on the genomic DNA templates of strains Zm6 and *pdc*. Expected lengths of the PCR products: 1.6 kb for intact *pdc* gene, 2.6 kb for gene with tet^r^ insert.**

Genomic DNA from Z. mobilis was isolated using a Promega Wizard Genomic DNA purification kit, following the manufacturer’s instructions, as in [13]. For PCR reaction, 10 µL of following assay mixture was used: 0.2 µL of each primer (10 µM stock solutions), 0.2 µL dNTP (10 mM stock), 0.8 µL of MgCl_2_ (25 mM stock), 1 µL of PCR buffer (10 x), 0.3 µL of genomic DNA preparation, 0.1 µL of ThermoScientific Dream Taq polymerase (5 U/µl), and 7.2 µL of water.

In termocycler, the initial denaturation phase of 3 min at 94 ^o^C was followed by 35 cycles with 30 sec denaturation at 94 ^o^C, 30 sec of primer annealing at 53 ^o^C, and 2 min 30 sec of extension phase at 72 ^o^C. The final extension phase was run for 10 min at 72 ^o^C.

**pdc1** – gtccagatt**ggatcc**aagcatcacttcgcag (GGATCC – BamHI)

**pdc2** – cttcagtgc**aagctt**cacgaccgatgaagc (AAGCTT – HindIII)

Pyruvate decarboxylase gene of *Z. mobilis* Zm6 (ATCC 29191) – ZZ6_1712:

atgagttatactgtcggtacctatttagcggagcggcttgtccagattggtctcaagcatcacttcgcagtcgcgggcgactacaacctcgtccttcttgacaacctgcttttgaacaaaaacatggagcaggtttattgctgtaacgaactgaactgcggtttcagtgcagaaggttatgctcgtgccaaaggcgcagcagcagccgtcgttacctacagcgttggtgcgctttccgcatttgatgctatcggtggcgcctatgcagaaaaccttccggttatcctgatctccggtgctccgaacaacaacgaccacgctgctggtcatgtgttgcatcatgctcttggcaaaaccgactatcactatcagttggaaatggccaagaacatcacggccgccgctgaagcgatttacaccccggaagaagctccggctaaaatcgatcacgtgatcaaaactgctcttcgcgagaagaagccggtttatctcgaaatcgcttgcaacactgcttccatgccctgcgccgctcctggaccggcaagtgcattgttcaatgacgaagccagcgacgaagcatccttgaatgcagcggttgacgaaaccctgaaattcatcgccaaccgcgacaaagttgccgtcctcgtcggcagcaagctgcgcgctgctggtgctgaagaagctgctgttaaattcaccgacgctttgggcggtgcagtggctactatggctgctgccaagagcttcttcccagaagaaaatgccaattacattggtacctcatggggcgaagtcagctatccgggcgttgaaaagacgatgaaagaagccgatgcggttatcgctctggctcctgtcttcaacgactactccaccactggttggacggatatccctgatcctaagaaactggttctcgctgaaccgcgttctgtcgttgtcaacggcattcgcttccccagcgttcatctgaaagactatctgacccgtttggctcagaaagtttccaagaaa**accggt**tctttggacttcttcaaatccctcaatgcaggtgaactgaagaaagccgctccggctgatccgagtgctccgttggtcaacgcagaaatcgcccgtcaggtcgaagctcttctgaccccgaacacgacggttattgctgaa**accggt**gactcttggttcaatgctcagcgcatgaagctcccgaacggtgctcgcgttgaatatgaaatgcagtggggtcacattggttggtccgttcctgccgccttcggttatgccgtcggtgctccggaacgtcgcaacatcctcatggttggtgatggttccttccagctgacggctcaggaagttgctcagatggttcgcctgaaactgccggttatcatcttcttgatcaataactatggttacaccatcgaagttatgatccatgatggtccgtacaacaacatcaagaactgggattatgccggtctgatggaagtgttcaacggtaacggtggttatgacagcggtgctgctaaaggcctgaaggctaaa**accggt**ggcgaactggcagaagctatcaaggttgctctggcaaacaccgacggcccaaccctgatcgaatgcttcatcggtcgtgaagactgcactgaagaattggtcaaatggggtaagcgcgttgctgccgccaacagccgtaagcctgttaacaagctcctctag

**Figure S2. The ORF of pyruvate decarboxylase (*pdc*)gene, and the primers pdc1 (yellow) and pdc2 (green) with engineered restriction sites, used for amplification.**

Insertional knock-out of the pyruvate decarboxylase was constructed [12] as follows. The amplified *pdc* fragment of 1.6 kb length contained 3 sites of AgeI (red, underlined). This fragment was cloned between BamHI and HindIII sites of the MCS of plasmid vector pGEM3Zf(+), and digested with AgeI. In place of the 0.5 kb, excised by AgeI digestion, a 1.5 kb DNA fragment carrying the tetracycline resistance determinant with engineered terminal AgeI sites was inserted. The resulting construct pGEMpdc:tet was used for transformation, and selection of homologous recombinants was carried out on plates with tetracycline. The primer pair pdc1 and pdc2 should give a 2.6 kb PCR product on the genomic DNA template of the *pdc* knock-out strain.
